# Gd(iii)–Pt(iv) theranostic contrast agents for tandem MR imaging and chemotherapy†

**DOI:** 10.1039/c9sc05937g

**Published:** 2020-01-28

**Authors:** Casey J. Adams, Thomas J. Meade

**Affiliations:** Departments of Chemistry, Molecular Biosciences, Neurobiology, and Radiology, Northwestern University Evanston Illinois 60208 USA tmeade@northwestern.edu

## Abstract

Pt(iv) prodrugs have emerged as versatile therapeutics for addressing issues regarding off-target toxicity and the chemoresistance of classic Pt(ii) drugs such as cisplatin and carboplatin. There is significant potential for Pt(iv) complexes to be used as theranostic agents, yet there are currently no reported examples of Gd(iii)–Pt(iv) agents for simultaneous MR imaging and chemotherapy. Here we report the synthesis, characterization, and *in vitro* efficacy of two Gd(iii)–Pt(iv) agents, **GP1** and **GP2**. Both agents are water soluble and stable under extracellularly relevant conditions but are reduced under intracellular conditions. Both are cytotoxic in multiple cancer cell lines, cell permeable, and significantly enhance the *T*_1_-weighted MR contrast of multiple cell lines. Thus, **GP1** and **GP2** are promising agents for tandem MR imaging and chemotherapy and provide a versatile platform through which future Gd(iii)–Pt(iv) agents can be developed.

## Abstract 2

The first example of Gd(iii)–Pt(iv) theranostic agents that are intracellularly reduced to provide MR contrast enhancement with simultaneous Pt(ii) chemotherapy.

## Graphical abstract

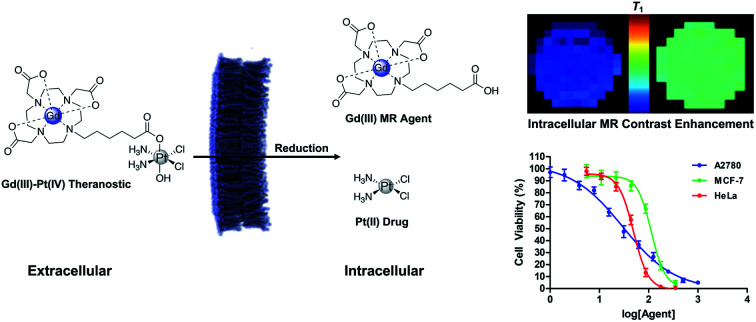

## Introduction

For decades, Pt(ii) chemotherapeutics have been fundamental tools for the treatment of solid tumors.^1–3^ Although there have been extensive efforts to develop new Pt-based chemotherapeutics, there are currently only three approved by the FDA: cisplatin, carboplatin, and oxaliplatin.^4–7^ All FDA approved Pt chemotherapeutics are Pt(ii) square planar complexes that cross-link DNA, ultimately leading to apoptosis in fast-dividing cells.^1,8^ This mechanism of action makes them highly effective at treating solid tumors. Even so, they have significant off-target toxicity that can result in a number of serious side effects including renal failure, hearing loss, and myelosuppression.^1–5^ Pt(ii) chemotherapeutics are also susceptible to chemoresistance. This stems from multiple mechanisms, including decreased drug accumulation, cytosolic sequestration, and resistance to DNA damage.^8–12^ Despite issues with toxicity and chemoresistance, Pt(ii) complexes are used in nearly 50% of all chemotherapy regimens, making them some of the most widely used cancer drugs available.^13^

A number of approaches have been attempted to mitigate off-target toxicity and chemoresistance of Pt(ii) drugs. This includes using tumor-targeting groups, nanoconstructs, and selective release mechanisms.^14–16^ In recent years, Pt(iv) prodrugs have become a promising approach to alleviate off-target toxicity and reduce chemoresistance.^14–19^ Pt(iv) complexes are octahedral and inert compared to their Pt(ii) square planar analogues. In an oxidizing extracellular environment Pt(iv) complexes remain inert. They can be reduced intracellularly to Pt(ii), triggering the dissociation of a reactive Pt(ii) drug from its axial ligands.^15–19^ A variety of groups can be incorporated as axial ligands to allow for tumor targeting, combination therapy, bioimaging, and controlled reduction of the Pt(iv).^20–31^ Sessler and coworkers were the first to demonstrate how Gd(iii) complexes can be used synergistically with Pt(iv) prodrugs. Their Gd(iii)-texaphyrin complexes have been used to increase tumor localization and mediate the reduction of Pt(iv) to Pt(ii).^30,31^ However, we believe there is a significant opportunity to investigate Gd(iii)–Pt(iv) mixed metal complexes for applications as theranostic agents.

Several examples of theranostic Pt(iv) prodrug complexes for dual chemotherapy and fluorescence imaging have been reported.^24–27^ Some of these probes can provide important information regarding drug delivery and subsequent reduction of the Pt(iv) complex *in vitro*. However, *in vivo* fluorescence imaging has limited clinical utility.^32,33^ The primary modality for imaging tumors is MRI, which allows for whole body, non-invasive imaging with excellent soft tissue contrast and spatial resolution.^32–36^ While a few examples of Gd(iii)–Pt(ii) theranostics have appeared,^37–39^ to our knowledge there are no reported examples of Pt(iv) prodrugs containing a Gd(iii) complex for contrast-enhanced MR imaging either *in vitro* or *in vivo*.

**Scheme 1:**
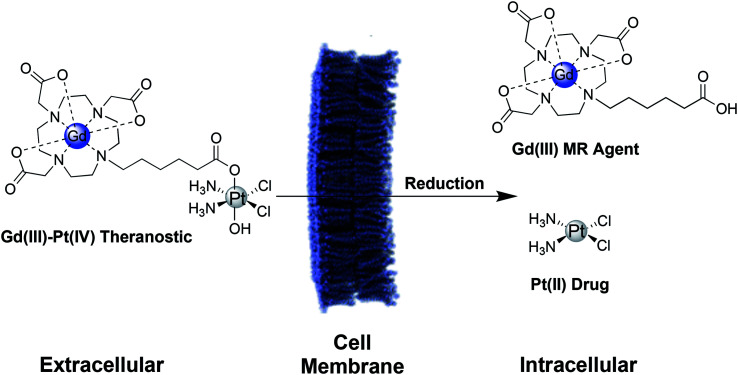
Schematic of a Gd(iii)–Pt(iv) theranostic agent. In the oxidizing extracellular environment, the agent remains in the Pt(iv) oxidation state.^15^ Upon entering the cell, the agent is reduced to Pt(ii), simultaneously releasing the therapeutic Pt(ii) drug and a Gd(iii) MR contrast agent.

Here, we describe two Gd(iii)–Pt(iv) theranostic agents that were synthesized by coupling a Gd(iii) MR contrast agent axially to cisplatin and carboplatin-based Pt(iv) complexes. These agents are water soluble, cell permeable, and oxidatively stable, but are reduced under biologically relevant intracellular conditions to release the toxic Pt(ii) payload and the Gd(iii) MR agent (Scheme 1). These complexes are designed for *intracellular* contrast enhancement of cancer cells, whereas typical Gd(iii) contrast agents are limited to the extracellular space surrounding tumors.^34,40^ This Gd(iii)–Pt(iv) platform possesses a second axial site that can be used to couple targeting groups for tumor specificity, drugs to combat chemoresistance, or fluorophores for multimodal imaging and validation.

## Results and discussion

### Synthesis and purification of the agents

**Fig. 1:**
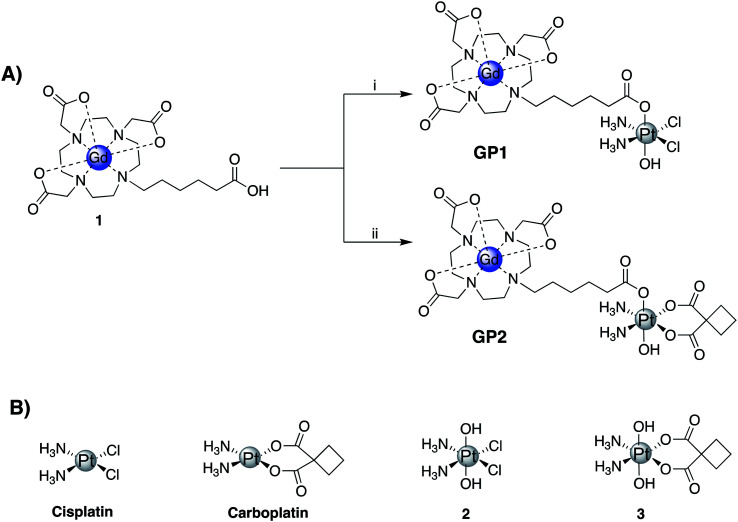
(A) Synthetic scheme of **GP1** and **GP2**: (i) TBTU (1 equiv.), triethylamine (1 equiv.), **2** (1.5 equiv.), DMSO, 45 °C, 12 h, 44%; (ii) TBTU (1 equiv.), triethylamine (1 equiv.), **3** (1.5 equiv.), DMSO, 45 °C, 12 h, 56%. (B) Structures of cisplatin, carboplatin, and the corresponding Pt(iv) complexes **2** and **3**.

Two Gd(iii)–Pt(iv) agents, **GP1** and **GP2** were synthesized by coupling a Gd(iii) complex, **1**, with Pt(iv) complexes **2** and **3** respectively (Fig. 1A). Complexes **1**, **2**, and **3** were all synthesized following literature protocols.^41–43^ The structures of **GP1** and **GP2** are found in Fig. 1A. Both agents were purified by preparatory HPLC, characterized by HPLC-MS (see ESI†) and analyzed by ICP-MS to ensure the Gd : Pt ratio was 1 : 1.

### Relaxivity measurements

The *T*_1_ relaxivity (*r*_1_) and *T*_2_ relaxivity (*r*_2_) of **1**, **GP1**, and **GP2** were measured at both low (1.41 T) and high (7 T) magnetic field strength in phosphate-buffered saline (PBS) to quantify how well they behave as MR contrast agents. Relaxivity values are summarized in Table 1. At 1.41 T, **1** had an *r*_1_ of 4.1 mM^−1^ s^−1^ and an *r*_2_ of 4.6 mM^−1^ s^−1^. At 7 T, the *r*_1_ had a slight increase while the *r*_2_ significantly increased, as expected at high field. The relaxivity values for **1** are well within the range of what is expected for a small molecule Gd(iii) complex.^34^ At 1.41 T, **GP1** and **GP2** had an *r*_1_ of 7.0 and 8.8 mM^−1^ s^1^ and *r*_2_ of 7.5 and 10.7 mM^−1^ s^−1^ respectively. At 7 T, there was little change in *r*_1_ for both agents, while both *r*_2_ values increased, as expected.

**Table 1:** *r*_1_ and *r*_2_ of **1**, **GP1**, and **GP2** at low (1.41 T) and high (7 T) magnetic field strength in PBS

Agent	*r*_1_ (mM^−1^ s^−1^)		*r*_2_ (mM^−1^ s^−1^)	
	1.41 T	7 T	1.41 T	7 T
**1**	4.1	4.7	4.6	6.9
**GP1**	7.0	7.1	7.5	10.7
**GP2**	8.8	8.5	9.2	12.4

Compared to **1**, both **GP1** and **GP2** had significantly increased relaxivity (both *r*_1_ and *r*_2_). This is possibly due to an increase in the rotational correlation time (*τ*_R_) or a change in the inner sphere hydration number (*q*) of Gd(iii). The differences in relaxivity of **GP1** and **GP2** compared to **1** offers the possibility of monitoring reduction of the agents by MR at both high and low field strength. To test this, **GP1**, **GP2**, and **1** were incubated in 5 mM GSH in PBS and the relaxivity was measured (see ESI†). The relaxivity of each converged to the same value (*r*_1_ of 4.4, 4.5, and 4.6 mM^−1^ s^−1^ for **1**, **GP1**, and **GP2** respectively), indicating the same Gd(iii) species was formed after reduction. The significant change in *r*_1_ upon reduction of **GP1** and **GP2** theoretically could be used to monitor *intracellular* reduction by MR. However, further testing needs to be done to determine if these agents are suitable for this application.

### Stability and reduction of the Gd(iii)–Pt(iv) complexes

Though most Pt(iv) complexes (see **2** and **3** in Fig. 1) are insoluble in most solvents, both **GP1** and **GP2** are readily soluble in aqueous solutions. To ensure both agents remain stable in aqueous media under various conditions, they were dissolved in PBS, two types of cell culture media (MEM and RPMI-1640), pH 5 H_2_O, and porcine live esterase (PLE) in PBS and monitored over time by HPLC-MS.

**Fig. 2:**
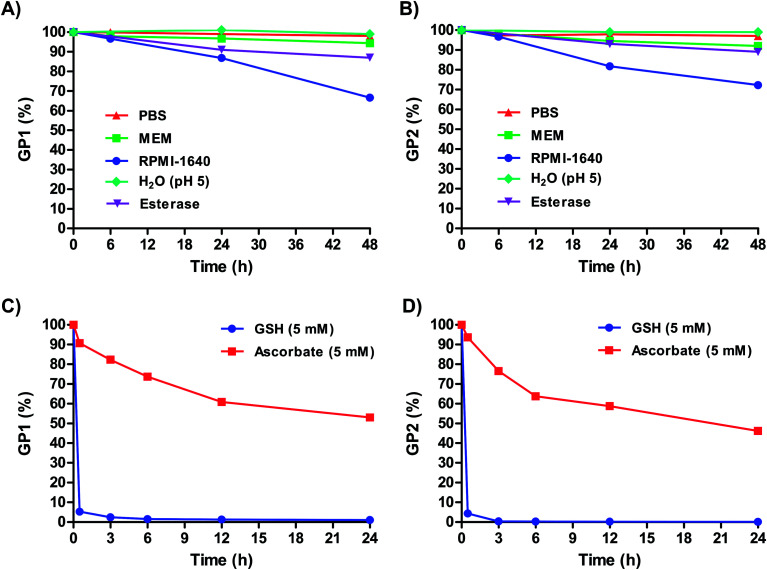
Stability of (A) **GP1** and (B) **GP2** in PBS, MEM, RPMI-1640, pH 5 H_2_O, and porcine liver esterase (PLE) in PBS. The observed partial reduction over long periods of time in RPMI-1640 is likely a result of glutathione (GSH) in the media. Both agents are highly stable in PBS, MEM, and pH 5 H_2_O over extended periods of time while PLE resulted in slight decrease in stability. Stability of (C) **GP1** and (D) **GP2** in 5 mM GSH and 5 mM ascorbate. **GP1** and **GP2** are both rapidly reduced by intracellular concentrations of GSH.

Fig. 2A and B shows that over 48 hours, **GP1** and **GP2** both remained completely stable in PBS and pH 5 H_2_O. This suggests that both agents can remain intact even in the most acidic conditions in cells, such as in lysosomes (pH 4.5–5).^44^ Both agents also remained highly stable in MEM (≥92% intact) at 48 hours. **GP1** and **GP2** were mostly stable in the presence of PLE, and the slight decrease in stability that was observed (∼10%) occurred on a much slower timescale than the reduction of both agents by GSH (Fig. 2C and D). It is unlikely that esterase cleavage is a competing intracellular dissociation mechanism.

In RPMI-1640, 33% of **GP1** and 28% of **GP2** were reduced to Pt(ii) within 48 hours. Unlike MEM, which contains no glutathione (GSH), RPMI-1640 has 0.003 mM GSH. Though this is an extracellularly relevant concentration of GSH (0.002–0.02 mM),^45,46^ a minority of **GP1** and **GP2** are reduced over long periods of time. However, it is important to note that at 6 hours, both agents are ≥96% intact (*i.e.*, they remain in the Pt(iv) oxidation state). *In vivo*, agent that does not get into cells within this time frame will likely be renally cleared before it is reduced. Therefore, within the time frame relevant for *in vivo* experiments, **GP1** and **GP2** stay intact and stable in extracellularly relevant conditions.

Under reducing conditions both agents rapidly converted to a monomeric Gd(iii) complex and a Pt(ii) drug. Each agent was incubated with 5 mM GSH and 5 mM ascorbate at 37 °C to mimic intracellular conditions. Fig. 2C and D shows that both agents are rapidly reduced by GSH at these concentrations (≥94% reduction in less than an hour). As expected, **GP1** and **GP2** are also reduced by ascorbate, albeit it at a much slower rate. This is not surprising, as ascorbate is a weaker reducing agent. It is clear from these results that under conditions similar to those inside cells, both agents are reduced, releasing a Gd(iii) contrast agent and Pt(ii) chemotherapeutic.

### Cytotoxicity of the agents

To assess the cytotoxicity of **GP1** and **GP2***in vitro*, cell viability assays were performed to determine the IC_50_ concentrations in three cancer cell lines (A2780, HeLa, and MCF-7). Table 2 summarizes the IC_50_ concentrations of both agents compared to those of cisplatin and carboplatin. Cells were incubated with cisplatin and **GP1** for 48 h and carboplatin and **GP2** for 72 h to compensate for the inherent decreased potency of carboplatin compared to cisplatin. **GP1** had an IC_50_ of 29.8 μM in A2780 cells, 49.3 μM in HeLa cells, and 113 μM in MCF-7 cells. For all cell lines, **GP1** was less toxic than cisplatin but followed the same trend of highest toxicity in A2780 cells and least toxic in MCF-7 cells. **GP1** releases cisplatin intracellularly, therefore the apparent difference in toxicity of the two is likely due to decreased cell permeability of **GP1** caused by the Gd(iii) complex. However, because **GP1** is inert in the extracellular matrix, higher doses can be safely administered to account for the decreased cell permeability.

**Table 2:** IC_50_ concentrations of cisplatin, carboplatin, **GP1**, and **GP2** in various cell lines

Complex	IC_50_ (μM)		
	A2780	HeLa	MCF-7
Cisplatin	7.6 ± 2.3	14.7 ± 1.2	22.4 ± 2.0
Carboplatin	15.3 ± 5.8	71.2 ± 4.9	124 ± 4
**GP1**	29.8 ± 2.5	49.3 ± 1.3	113 ± 4
**GP2**	55.0 ± 2.9	258 ± 5.0	382 ± 6

**GP2** had an IC_50_ of 55.0 μM in A2780 cells, 258 μM in HeLa cells, and 382 μM in MCF-7 cells. The decreased toxicity of **GP2** compared to **GP1** is expected because carboplatin is known to be significantly less reactive than cisplatin. **GP2** was similarly less toxic than carboplatin, but followed the same trend of highest toxicity in A2780 cells and lowest toxicity in MCF-7 cells. The decreased toxicity compared to carboplatin is again attributed to decreased cell permeability due to the presence of the Gd(iii) complex.

These results demonstrate that **GP1** and **GP2** are cytotoxic in three different cancer cell lines. Both agents show similar trends in toxicity as their Pt(ii) analogues, which suggests that upon entering the cells they are reduced and behave like typical Pt(ii) chemotherapeutics.

### Accumulation of the Gd(iii)–Pt(iv) agents in cells

Accumulation of **GP1** and **GP2** in cells was measured by concentration-dependent uptake experiments in A2780 and HeLa cells. Individual concentration-dependent uptake graphs are found in the ESI.†Table 3 compares the accumulation of Gd and Pt in A2780 and HeLa cells that were incubated with **GP1**, **GP2**, cisplatin, and carboplatin near their respective IC_50_ concentrations. For comparison, both cell lines were dosed with complex **1** at similar concentrations as **GP1** and **GP2**. The results of these uptake experiments demonstrate several important points. First, the accumulation of Gd in both cell lines for both agents is higher than what is typically considered the amount necessary for detection by MR (high μM concentrations).^32,33^ This suggests that **GP1** and **GP2** can significantly enhance intracellular MR contrast. Typical Gd(iii) complexes alone are not cell permeable, therefore the Pt(iv) complexes likely make it possible for **GP1** and **GP2** to get into cells. This is evidenced by the significant increase in cellular uptake of **GP1** and **GP2** compared to complex **1** in both cell lines.

**Table 3:** Accumulation of Gd and Pt in A2780 and HeLa cells when incubated with **GP1**, **GP2**, cisplatin, and carboplatin near their IC_50_ concentrations and **1** at 100 μM for 24 h

Complex	Cell accumulation (fmol per cell)			
	A2780 cells		HeLa cells	
	Gd	Pt	Gd	Pt
**GP1**	2.5 ± 0.3	0.20 ± 0.03	5.7 ± 1.9	0.17 ± 0.01
**GP2**	1.4 ± 0.2	0.38 ± 0.1	1.1 ± 0.2	0.30 ± 0.06
**1**	0.26 ± 0.02	N/A	0.21 ± 0.01	N/A
Cisplatin	N/A	0.21 ± 0.02	N/A	0.27 ± 0.02
Carboplatin	N/A	0.22 ± 0.02	N/A	0.60 ± 0.11

Second, intracellular Pt levels at the IC_50_ concentrations were similar between **GP1** and cisplatin and **GP2** and carboplatin. From a therapeutic standpoint, the agents behave like cisplatin and carboplatin once they enter cells and are reduced from Pt(iv) to Pt(ii). These results support that the higher IC_50_ concentrations of **GP1** and **GP2** compared to cisplatin and carboplatin are caused by decreased cell-permeability, not a decrease in intracellular toxicity. It is clear that both **GP1** and **GP2** penetrate cells well enough to provide sufficient MR-Gd(iii) concentrations for imaging and therapeutically relevant Pt concentrations in cells at low incubation concentrations.

Finally, there is a significant preferential accumulation of Gd compared to Pt from both agents in both cell lines. The difference in accumulation of the two is possible because they dissociate from one another after the intracellular reduction of Pt(iv) to Pt(ii). For **GP1**, a 13-fold higher accumulation of Gd than Pt in A2780 cells and 34-fold higher Gd(iii) accumulation in HeLa cells was observed. For **GP2**, Gd accumulation was 3.7-fold higher in both A2780 and HeLa cells. These results suggest that Gd is effluxed to a lesser extent than Pt. When **GP1** and **GP2** are reduced intracellularly, the cell impermeable charged Gd(iii) complex is likely prevented from exiting the cells as readily as the cell permeable Pt(ii) complexes. This is of particular consequence for the ability of **GP1** and **GP2** to act as MR contrast agents because higher cellular amounts of Gd(iii) increase MR contrast.

**Fig. 3:**
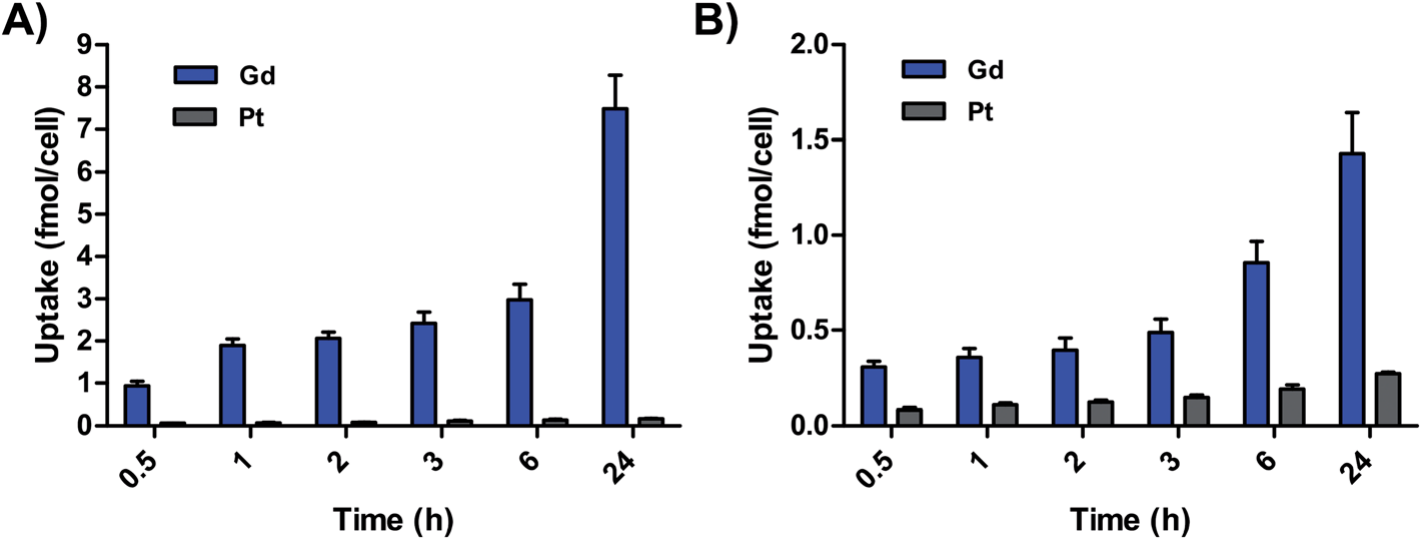
(A) Accumulation of Gd and Pt in A2780 cells incubated with 65 μM **GP1** over time. (B) Accumulation of Gd and Pt in A2780 cells incubated with 62.5 μM **GP2** over time. In both cases, accumulation of Gd was significantly higher than Pt.

Cell uptake of **GP1** and **GP2** was additionally measured in a time-dependent manner in A2780 cells. Fig. 3 demonstrates that the accumulation of both Gd and Pt from both agents is directly proportional to time. In these experiments, higher accumulation of Gd compared to Pt was observed at every timepoint. Initial uptake of **GP1** and **GP2** from 0–30 min was fast compared to uptake from 0.5–24 h. Fast uptake and intracellular reduction can explain why there was significantly more Gd than Pt even at the 30 min timepoint. For both agents, the Gd : Pt ratio in cells continues to increase over time, which is consistent with a higher rate of efflux of Pt. These results also suggest MR-relevant amounts of Gd from both agents accumulate in cells within a few hours. This is promising for the prospect of imaging within the time frame relevant for *in vivo* experiments.

### MR imaging of A2780 and HeLa cell pellets

To quantify the ability of **GP1** and **GP2** to enhance MR contrast intracellularly, MR cell pellet imaging experiments were performed. A2780 and HeLa cells were incubated with IC_50_ concentrations of **GP1** and **GP2** for 6 hours then imaged in a 7 T MR scanner. The relaxation rate (*R*_1_), which is defined as 1/*T*_1_ and is directly proportional to MR contrast, of each cell pellet was determined and compared to control cells that were untreated, incubated with 100 μM complex **1**, or incubated with IC_50_ concentrations of cisplatin.

**Fig. 4:**
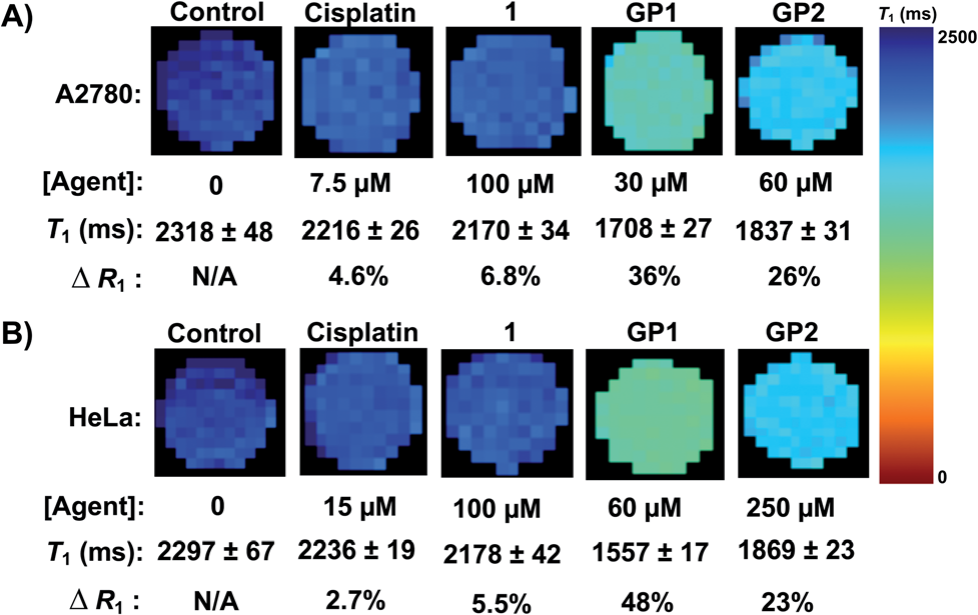
(A) MR imaging of A2780 cell pellets at 7 T. IC_50_ concentrations of **GP1** and **GP2** significantly enhanced the intracellular contrast of A2780 cells (increase in *R*_1_) compared to the untreated control cells while **1** and cisplatin had a minimal effect. (B) MR imaging of HeLa cell pellets at 7 T. IC_50_ concentrations of **GP1** and **GP2** significantly enhanced the intracellular contrast of HeLa cells compared to the untreated control cells while **1** and cisplatin had a minimal effect.

Fig. 4 demonstrates that **GP1** and **GP2** significantly enhance the intracellular MR contrast of both A2780 and HeLa cells when dosed at concentrations near the IC_50_. **GP1** increased the *R*_1_ by 36% in A2780 cells and 48% in HeLa cells compared to the untreated controls. **GP2** increased the *R*_1_ by 26% in A2780 cells and 23% in HeLa cells. Treating cells with similar concentrations of complex **1** and IC_50_ concentrations of cisplatin resulted in minimal increases in *R*_1_. This supports that the observed contrast enhancement by **GP1** and **GP2** is a result of the agents effectively accumulating in cells, something **1** cannot do alone. Furthermore, any physiological changes in the cells caused by the presence of a Pt(ii) drug have little effect on the MR contrast. Therefore, the contrast enhancement is a result of intracellular Gd(iii).

Notably, the cell pellet experiments were performed within a time frame relevant for *in vivo* MR imaging (6 hours). These results are very promising for the prospect of using Gd(iii)–Pt(iv) theranostics like **GP1** and **GP2** for *in vivo* MR imaging.

## Conclusions

We have described the synthesis, characterization and cellular uptake of two new Gd(iii)–Pt(iv) agents. **GP1** and **GP2** represent the first examples of Gd(iii)–Pt(iv) agents that are simultaneously MR contrast agents and are reduced to provide chemotherapy. Of the two agents, **GP1** is most promising as it exhibits greater cellular toxicity, higher intracellular accumulation of Gd(iii) and better MR contrast enhancement *in vitro*. Future work will focus on demonstrating the *in vivo* efficacy of these agents for both imaging and treatment.

## Conflicts of interest

There are no conflicts of interest to declare.

## Supplementary Material

SC-011-C9SC05937G-s001
