# Supplementary material for: Gd(iii)–Pt(iv) theranostic contrast agents for tandem MR imaging and chemotherapy
Source: Chem Sci. 2020 Jan 28;11(9):2524–30. doi: 10.1039/c9sc05937g (PMC8157322; doi:10.1039/c9sc05937g)
Supplement: SC-011-C9SC05937G-s001 [file SC-011-C9SC05937G-s001.pdf]

Electronic Supplementary Material (ESI) for Chemical Science.
This journal is © The Royal Society of Chemistry 2020

Gd(III)-Pt(IV) theranostic contrast agents for tandem MR imaging and chemotherapy

Casey J. Adams† and Thomas J. Meade*†

† Departments of Chemistry, Molecular Biosciences, Neurobiology, and Radiology, Northwestern University, Evanston, Illinois 60208, United States

Supporting Information

Experimental 2-7

HPLC-MS and ESI-MS of GP1 and GP2 8

Relaxivity Measurements 9

HPLC Chromatograms of GP1 and GP2 10-15

IC_{50} Curves of GP1 and GP2 16

Uptake Experiments 17-20

Experimental:

General Methods. Unless otherwise indicated, all reactions were performed under a nitrogen atmosphere using oven-dried glassware. Anhydrous solvents were used in all reactions and obtained from a J.C. Meyer solvent system (Laguna Beach, CA). Thin-layer chromatography (TLC) was performed on EMD 60 F254 silica gel plates. Standard grade 60 Å 230–400 mesh silica gel was used for normal-phase column chromatography. Unless otherwise stated, all silica gel columns were flashed with air. 1H and 13C NMR spectra were obtained on a Bruker 500 MHz Avance III NMR spectrometer. ESI-MS was performed on a Bruker AmaZon-SL spectrometer.

Cyclen was obtained from Strem Chemical, while all other reagents were purchased from Sigma Aldrich, Fisher Scientific, or TCI and used without purification. Analytical HPLC-MS was performed on an Agilent 1260 Infinity II HPLC system with an in-line Agilent 6120 Quad mass spectrometer. Semi-preparative HPLC was performed on an Agilent PrepStar 218 equipped with an Agilent 1260 Infinity diode array detector. HPLC purifications utilized deionized water (18.2 MΩ·cm) obtained from a Millipore Q-Guard System and HPLC grade MeCN, formic acid, and ammonium hydroxide (all obtained from Fisher Scientific).

Analytical HPLC-MS used an Atlantis C18 column (4.6 × 250 mm, 5 µm). Semipreparative HPLC used an Atlantis T3 C18 column (19 x 250 mm, 10 µm). **GP1** and **GP2** were purified using the following method: MeCN held at 0% for 5 min followed by a 25 min ramp to 75% followed by a 5 min ramp to 100%.

Synthesis of GP1. 9.0 mg **1** (0.015 mmol) were added to a 10 mL round bottom flask and dissolved in 3 mL of dry DMSO. 4.7 mg TBTU (0.015 mmol) and 2 µL triethylamine (0.015 mmol) were added and the solution was heated to 45 °C and stirred for 10 min. 7.3 mg **2** (0.023 mmol) were added and the reaction was stirred at 45 °C under nitrogen over night. The reaction mixture was lyophilized to dryness, dissolved in H_2O, and purified by semipreparative HPLC (retention

time: 13.8 min) in 44% yield. Analytical HPLC-MS trace of the purified product is found in Fig. S1A. ESI-MS m/z observed: 931.7, calculated: 931.1 $[M+H]^+$.

Synthesis of GP2. 5.5 mg **1** (0.009 mmol) were added to a 10 mL round bottom flask and dissolved in 2 mL of dry DMSO. 2.9 mg TBTU (0.009 mmol) and 1.2 μL triethylamine (0.009 mmol) were added and the solution was heated to 45 °C and stirred for 10 min. 5.5 mg **3** (0.014 mmol) were added and the reaction was stirred at 45 °C under nitrogen over night. The reaction mixture was then lyophilized to dryness, dissolved in H_2O, and purified by semipreparative HPLC (retention time: 15.3 min) in 56% yield. Analytical HPLC-MS trace of the purified product is found in Fig. S1B. ESI-MS m/z observed: 1003.4, calculated: 1003.19 $[M+H]^+$.

Relaxivity Measurements at 1.41 T. GP1 and **GP2**, and **1** were dissolved in 1 mL of PBS or 5 mM GSH in PBS. Each solution was serially diluted to make solutions of varying concentration. Relaxation times were measured on a Bruker mq60 NMR analyzer equipped with Minispec v 2.51 Rev.00/NT software (Bruker Biospin, Billerica, MA, USA) operating at 1.41 T (60MHz) and 37 °C. Measurements were made using an inversion recovery pulse sequence (T_1_ir_mb) using the following parameters: 4 scans per point, 10 data points, monoexponential curve fitting, phase cycling, 10 ms first pulse separation, and a recycle delay and final pulse separation ≥ 5 T_1. 10 μL aliquots of each solution were taken for ICP-MS analysis to determine the concentration of Gd(III).

Relaxivity Measurements at 7 T. GP1 and **GP2** and **1** were dissolved in 200 μL PBS or 5 mM GSH and each solution was serially diluted to make solutions of varying concentration. 25 μL of each solution were pipetted into flame sealed Pasteur pipettes. The pipette tips containing solution were scored, separated, and sealed with parafilm to make small capillaries containing

solution. These capillaries were imaged using a Bruker PharmaScan 7 T MR imaging spectrometer (Bruker BioSpin, Billerica, MA, USA). T_1 relaxation times were measured using a rapid-acquisition rapid-echo (RARE-VTR) T_1-map pulse sequence with static T_E (10 ms) and variable T_R (100, 200, 400, 500, 750, 1000, 2500, 7500, and 1000 ms) values. Imaging parameters were as follows: field of view (FOV) = 25 x 25 mm^2, matrix size (MTX) = 256 x 256, number of axial slices = 5, slice thickness (SI) = 1.0 mm, and averages (NEX) = 4. T_1 analysis was carried out using the image sequence analysis tool in Paravision 5.0 pl2 software (Bruker, Billerica, MA, USA) with monoexponential curve-fitting of image intensities of selected regions of interest (ROIs) for each axial slice.

Stability of GP1 and GP2 in various aqueous media. Aliquots of **GP1** and **GP2** were prepared from a stock with known mass checked by ICP-MS. An aliquot of each was dissolved in H_2O and analyzed by HPLC-MS using an Atlantis C18 column (4.6 × 250 mm, 5 μm) and the following method: MeCN held at 0% for 3 min followed by a 15 min ramp to 100% MeCN (**GP1** retention time: 10.9 min, **GP2** retention time: 11.6 min). Aliquots of both were additionally dissolved in PBS, MEM, RPMI-1640, pH 5 H_2O, PBS with 10 units/mL porcine liver esterase, 5 mM glutathione, and 5 mM sodium ascorbate, incubated in a shaker at 37 ^oC, and were analyzed by HPLC-MS at different time points using the same method. At each time point, the area of the peak of **GP1** or **GP2** was determined by integration and compared to the area of the peaks in H_2O. Difference in peak area was used as a means of determining the percentage of agent remaining in solution.

Cell Lines and Culture. A2780 cells were cultured using RPMI-1640 supplemented with 10% FBS. HeLa cells were cultured using phenol red free DMEM supplemented with 10% FBS. MCF-7 cells were cultured using phenol-red free MEM supplemented with 10% FBS. All three cell

lines were grown in a humidified incubator at 37 °C and 5% CO_2 and were harvested using 0.25% TrypLE. Cells were grown for 24 hours after plating before each experiment. All solutions were filtered through 0.2 μL sterile filters before use.

Viability Assays. A2780, HeLa, and MCF-7 cells were plated at a density of 3,000 cells per well (100 μL) in an opaque white 96-well plate. Cells were dosed with 100 μL of solutions of **GP1**, **GP2**, cisplatin, or carboplatin in MEM and incubated for 48 hours (**GP1** or cisplatin) or 72 hours (**GP2** or carboplatin). After incubation, 50 μL CellTiter-Glo 2.0 (Promega, Madison, WI) was added to each well and the assay was carried out following the manufacturer's protocol. Luminescence of the wells was measured using a Synergy H1 microplate reader (BioTek, Winooski, VT). Viability was determined by comparing luminescence readings of the cells treated with agent to untreated control cells.

Concentration-dependent Cell Uptake. A2780 and HeLa cells were plated at a density of 40,000 cells per well (500 μL) in a 24-well plate. Cells were incubated with **GP1**, **GP2**, **1**, cisplatin or carboplatin at varying concentrations in MEM (300 μL) for 24 hours. After 24 hours, the media was aspirated and the cells were washed twice with 500 μL of PBS, harvested, and centrifuged at 500 rpm for 5 minutes at 4° C. The media was aspirated and the cells were resuspended in 200 μL of media. A 50 μL aliquot was taken for cell counting using a Guava PCA system using the Guava Viacount protocol provided by the manufacturer. An additional 100 μL aliquot was used for ICP-MS analysis of Gd and Pt in the cells.

Cell fractionation experiments were performed using a cytosol/particulate rapid separation kit (BioVision, Milpitas, CA). Uptake experiments were performed in the same way, following the manufacturer's protocol after the first centrifugation. Gd and Pt content in each

fraction was determined by ICP-MS and the total uptake for a given set of cells was taken to be the sum of the cytosol and particulate fractions.

Time-dependent Cellular Uptake. A2780 cells were plated at a density of 40,000 cells per well (500 μL) in a 24-well plate. Cells were incubated with 300 μL of 65 μM **GP1** or 62.5 μM **GP2** in MEM for variable amounts of time (0.5, 1, 2, 3, 6, 24 h). At each timepoint, the media was aspirated and the cells were washed twice with 500 μL of PBS, harvested, and centrifuged at 500 rpm for 5 minutes at 4° C. The media was aspirated and the cells were resuspended in 200 μL of media. A 50 μL aliquot was taken for cell counting using a Guava PCA system using the Guava Viacount protocol provided by the manufacturer. An additional 100 μL aliquot was used for ICP-MS analysis of Gd and Pt in the cells.

MR Imaging of Cell Pellets at 7 T. A2780 and HeLa cells were grown to ~60% confluency in T-75 flasks. A2780 cells were dosed with 5 mL of 30 μM **GP1**, 60 μM **GP2**, 100 μM **1**, 7.5 μM, or vehicle (MEM) and HeLa cells were dosed with 5 mL of 60 μM **GP1**, 250 μM **GP2**, 100 μM **1**, 15 μM, or vehicle (MEM). Cells were incubated for 6 hours. After incubation, the media was aspirated and the cells were washed twice with 5 mL PBS, harvested, and centrifuged at 500 rpm for 5 min at 4 ^{o}C. The cells were resuspended in 1 mL of media and 950 μL of the suspension was added to flame-sealed Pasteur pipettes while the rest was used for cell counting and ICP-MS. The pipettes were centrifuged at 200 rpm for 5 minutes and were separated to form small capillaries containing the cell pellets. The capillaries were sealed with parafilm and imaged using a Bruker PharmaScan 7 T MR imaging spectrometer following the same imaging protocol previously described.

Cell Counting with a Guava EasyCyte Mini Personal Cell Analyzer (PCA) System. Aliquots of cell suspensions were mixed with the Guava ViaCount Reagent and allowed to stain for 5 minutes. The samples were vortexed for 20 seconds and cell count was determined via manual analysis using a Guava EasyCyte Mini PCA and ViaCount software. 1000 events were acquired for each sample and dilutions were performed to assure the cell count was in the optimal range for instrument performance (10-100 cells/μL). Performance of the instrument was assessed daily using Guava-Check Beads and the manufacturer's protocol and Daily Check software.

Quantification of Gadolinium and Platinum with Inductively Coupled Plasma Mass Spectrometry. Quantification of Gd and Pt was accomplished using ICP-MS of acid digested samples. Specifically, samples were digested in concentrated trace nitric acid (> 69%, Thermo Fisher Scientific, Waltham, MA, USA) and trace hydrochloric acid (> 34%, Thermo Fisher Scientific, Waltham, MA, USA) and placed at 65 °C for at least 4 hours to allow for complete sample digestion. Ultra-pure H_2O (18.2 MΩ·cm) was then added to produce a final solution of 2.0% nitric acid and 2.0% hydrochloric acid (v/v) in a total sample volume of 10 mL. Quantitative standards were made using a 10,000 µg/mL Gd elemental standard and a 1,000 ug/mL Pt elemental standard (Inorganic Ventures, Christiansburg, VA, USA) which were used to create a 200 ng/g mixed element standard and a 2 ng/g mixed element standard in 2.0% nitric acid and 2.0% hydrochloric acid (v/v) in a total sample volume of 50 mL. A solution of 2.0% nitric acid and 2.0% hydrochloric acid (v/v) was used as the calibration blank.

ICP-MS was performed on a computer-controlled (QTEGRA software) Thermo iCapQ ICP-MS (Thermo Fisher Scientific, Waltham, MA, USA) operating in STD mode and equipped with a ESI SC-2DX PrepFAST autosampler (Omaha, NE, USA). Internal standard was added inline using the prepFAST system and consisted of 1 ng/mL of a mixed element solution containing Bi, In, ^{6}Li, Sc, Tb, Y (IV-ICPMS-71D from Inorganic Ventures). Online dilution was also carried out

by the prepFAST system and used to generate a calibration curve consisting of 200, 100, 50, 20, 10, 2 ppb Gd and Pt and a calibration curve consisting of 2000, 1000, 500, 200, 100, and 20 ppt Gd and Pt. Each sample was acquired using 1 survey run (10 sweeps) and 3 main (peak jumping) runs (40 sweeps). The isotopes selected for analysis were $^{194,195}Pt$, $^{56,57}Gd$, and ^{115}In, ^{159}Tb, ^{209}Bi (chosen as internal standards for data interpolation and machine stability). Instrument performance is optimized daily through autotuning followed by verification via a performance report (passing manufacturer specifications).

Supplementary Figures:

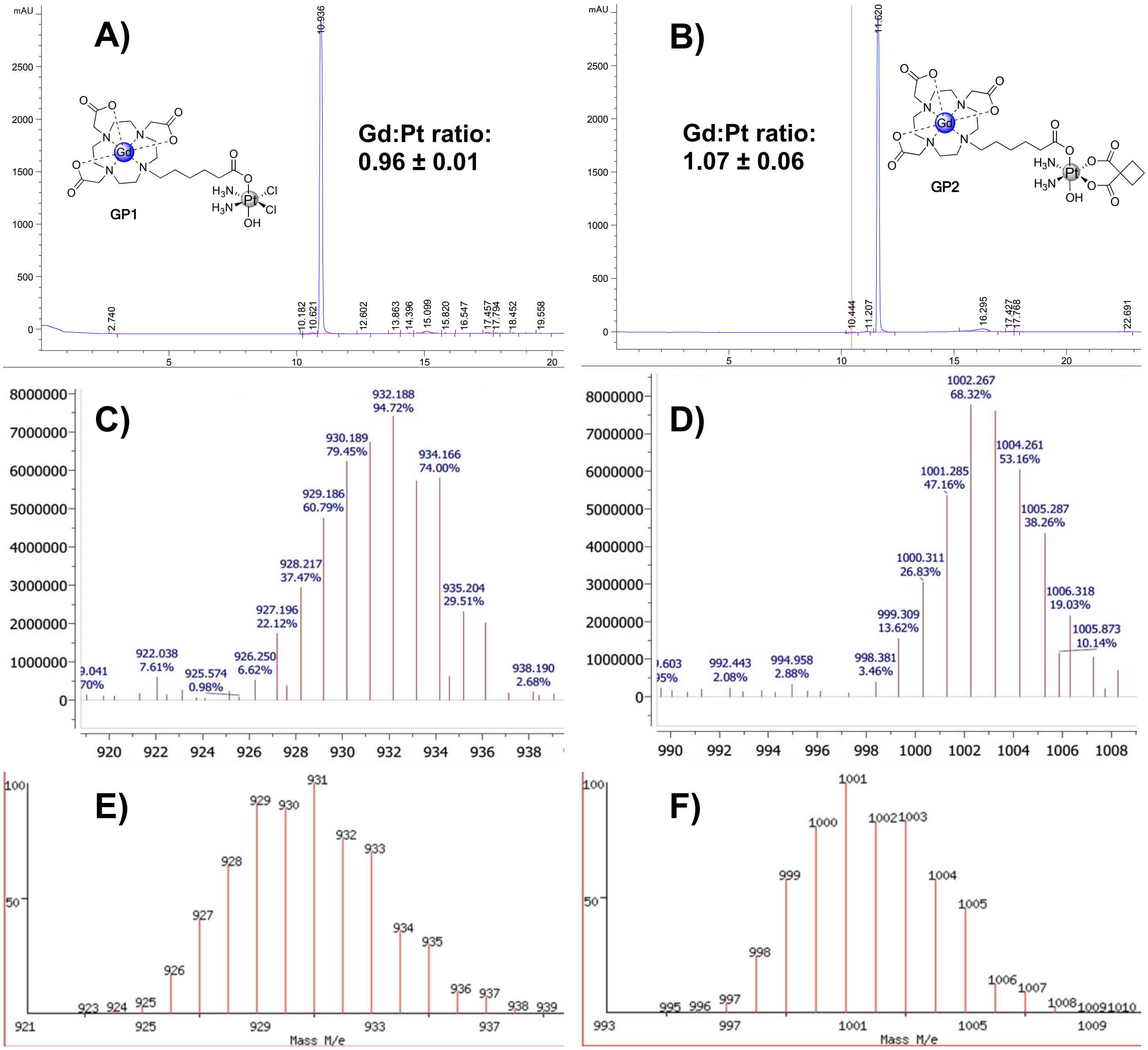

Fig. S1 (A) HPLC-MS trace of **GP1** purified by semipreparative HPLC. The reported Gd:Pt ratio was determined by ICP-MS analysis of all batches of **GP1**. (B) HPLC-MS of **GP2** purified by semipreparative HPLC. The reported Gd:Pt ratio was determined by ICP-MS analysis of all batches of **GP2**. (C) ESI-MS spectrum of purified **GP1**. (D) ESI-MS spectrum of purified **GP2**. (E) Predicted mass spectrum isotope pattern of **GP1**. (F) Predicted mass spectrum isotope pattern of **GP2**.

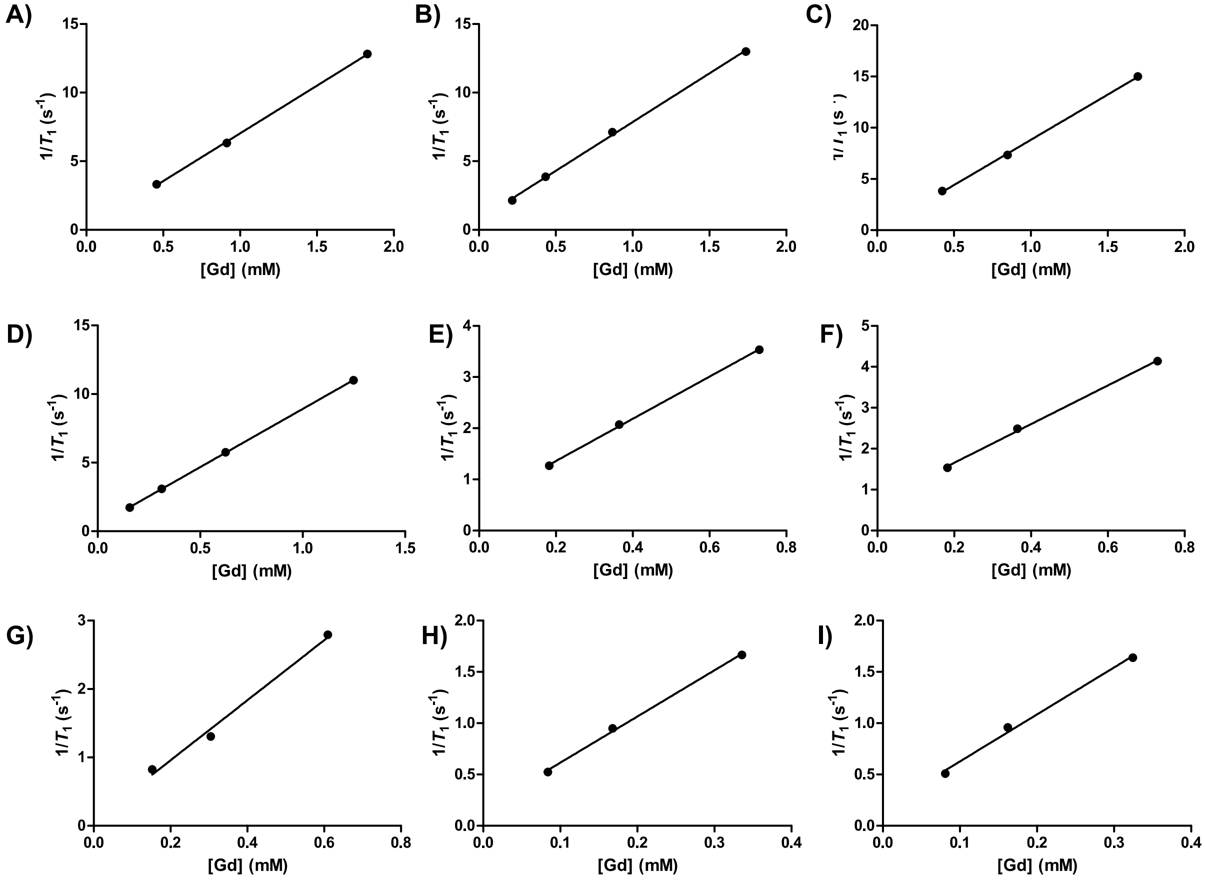

Fig. S2 Graph of r_1 of (A) **GP1** measured at 1.41 T (B) **GP1** measured at 7 T (C) **GP2** measured at 1.41 T (D) **GP2** measured at 7 T (E) **1** measured at 1.41 T (F) **1** measured at 7 T. All measurements were taken in PBS at 37 °C (G) **1** measured at 1.41 T with 5 mM GSH (H) **GP1** measured at 1.41 T with 5 mM GSH and (I) **GP2** measured at 1.41 T with 5 mM GSH.

Table S1. r_1 of **GP1**, **GP2**, and **1** in PBS with 5 mM glutathione at 37 °C

	GP1 (5 mM GSH)	GP2 (5 mM GSH)	1 (5 mM GSH)
r_1 ($mM^{-1}s^{-1}$) at 1.41 T	4.5	4.6	4.4
r_2 ($mM^{-1}s^{-1}$) at 1.41 T	5.1	5.0	4.9

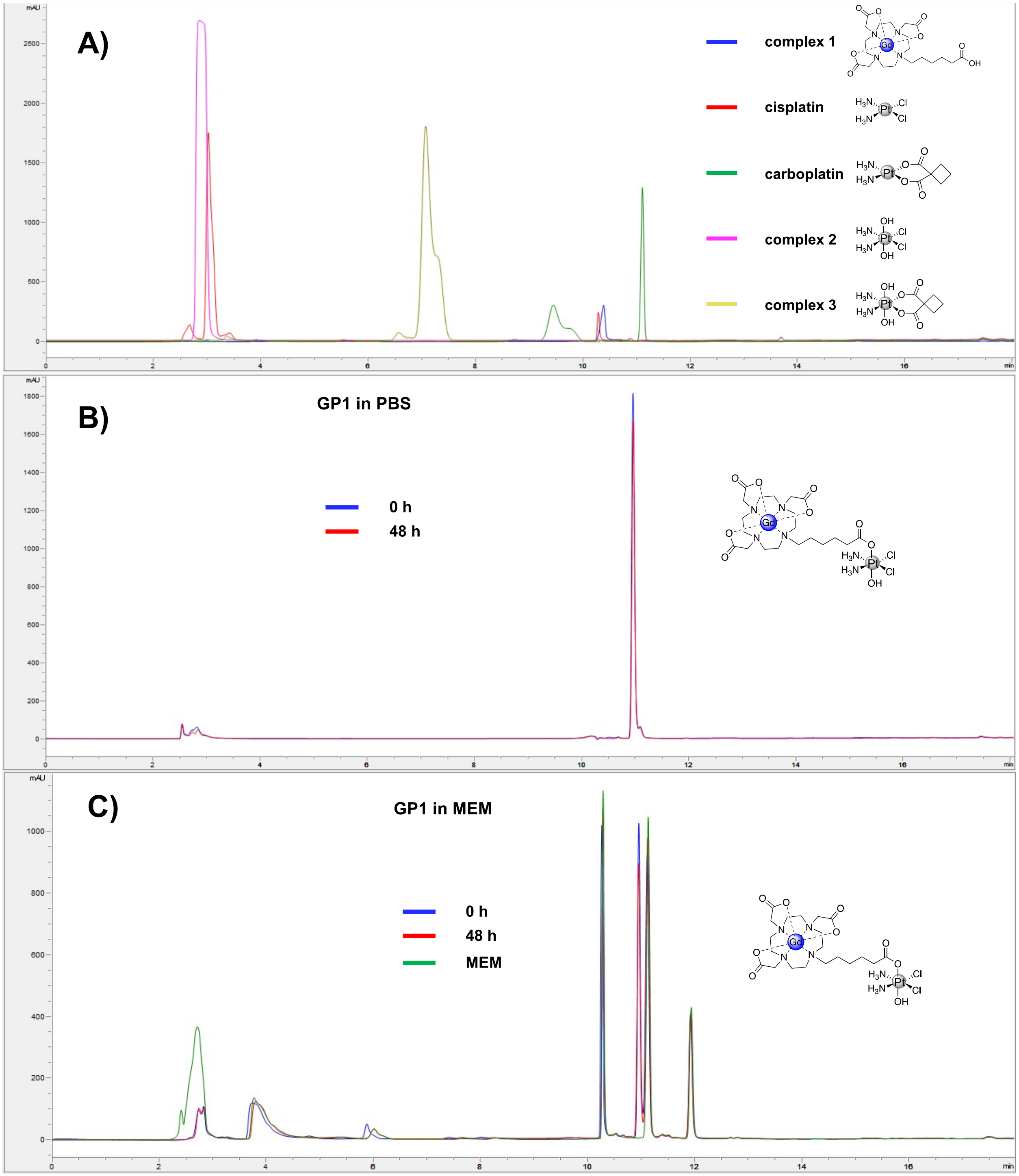
A)
complex 1
cisplatin
carboplatin
complex 2
complex 3
B)
GP1 in PBS
0 h
48 h
C)
GP1 in MEM
0 h
48 h
MEM

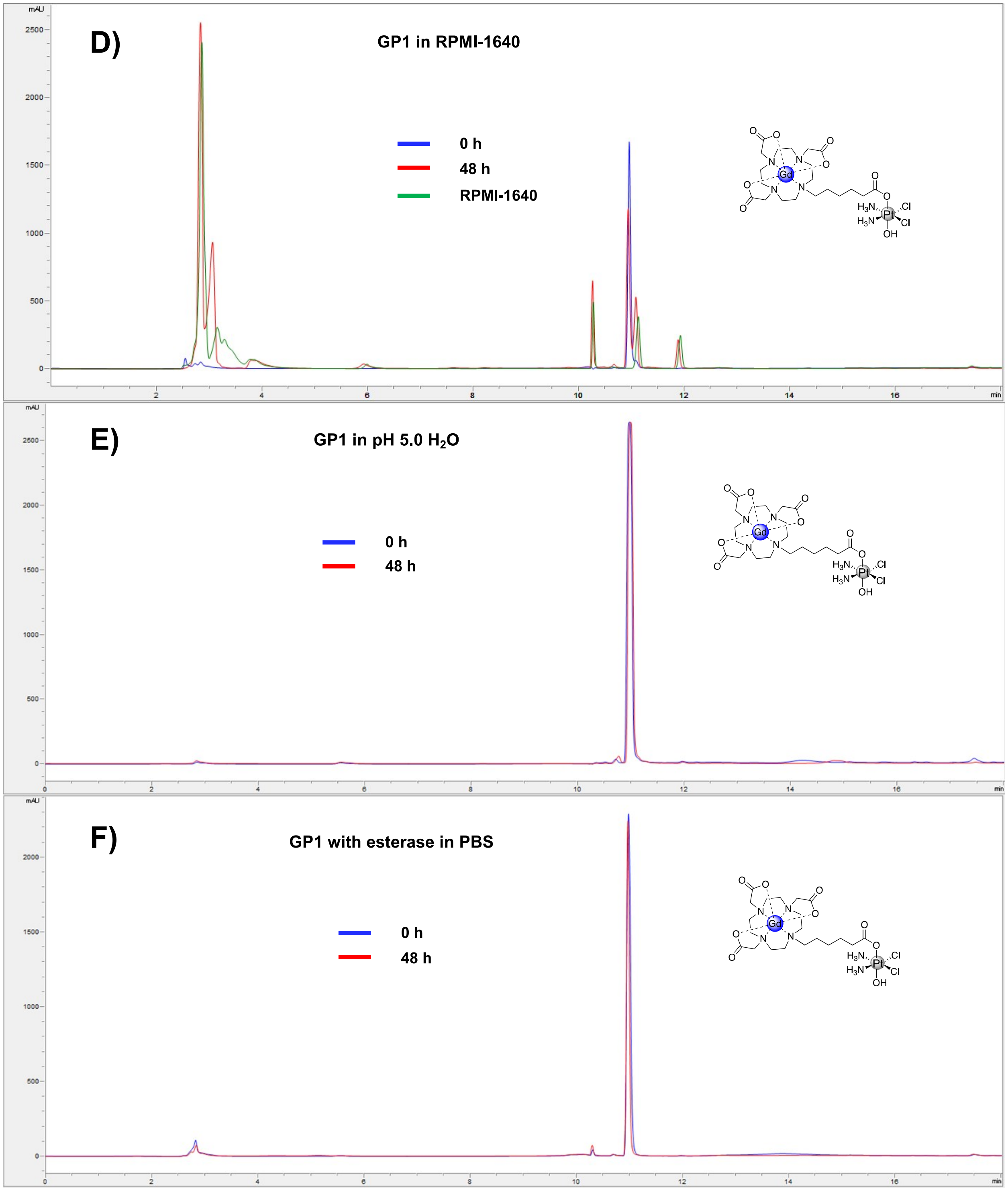
D)
GP1 in RPMI-1640
0 h
48 h
RPMI-1640
E)
GP1 in pH 5.0 H_2O
0 h
48 h
F)
GP1 with esterase in PBS
0 h
48 h
mAU
min

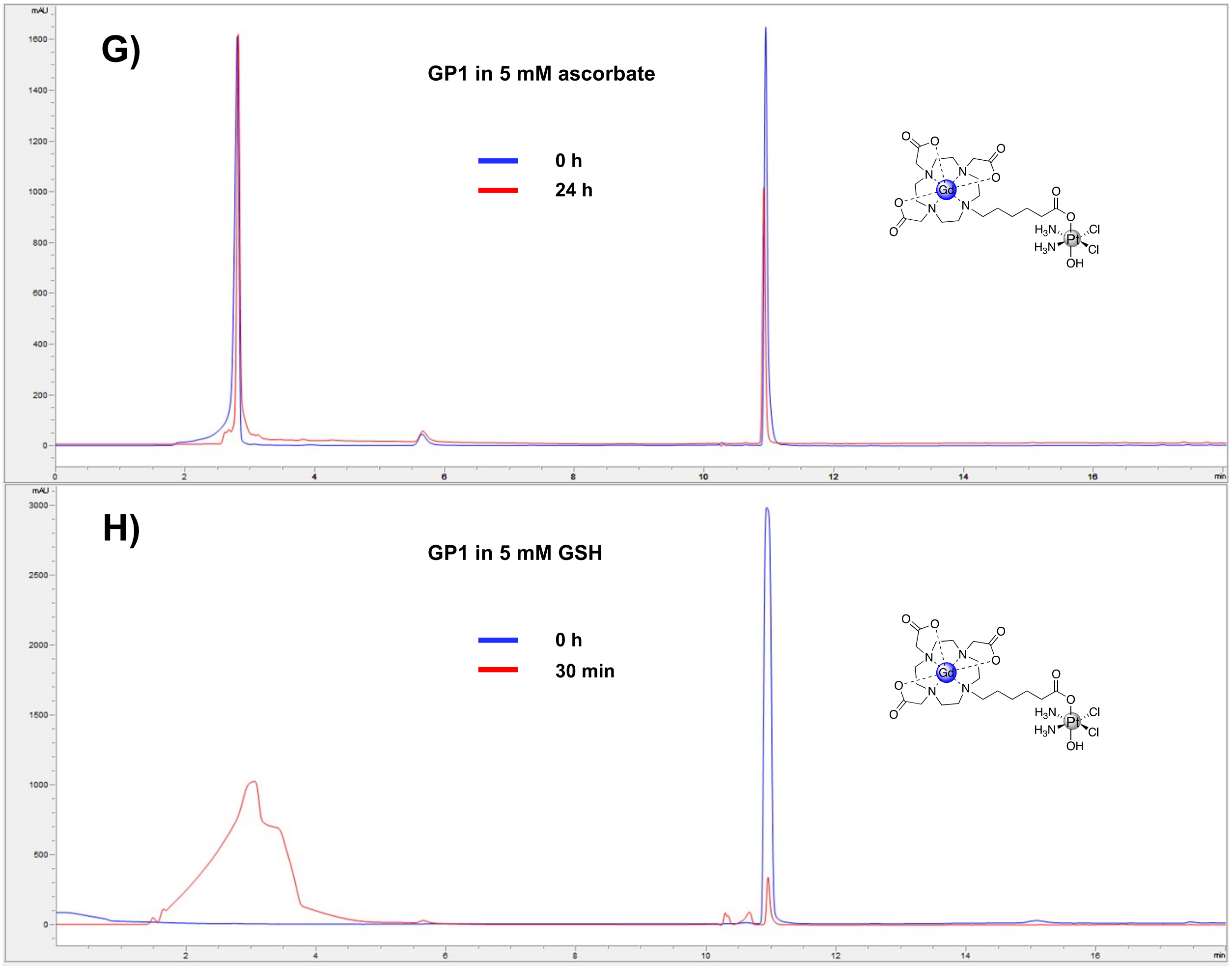

Fig. S3 Analytical HPLC chromatograms of (A) cisplatin, carboplatin, **1**, **2**, and **3** in H_2O (B) **GP1** in PBS over time (C) **GP1** in MEM over time (D) **GP1** in RPMI-1640 over time (E) **GP1** in pH 5 H_2O over time (F) **GP1** in PBS with porcine liver esterase (PLE) over time (G) **GP1** in 5 mM ascorbate over time and (H) **GP1** in 5 mM glutathione (GSH) over time. All HPLC runs used the same method (0-3 min 100% H_2O followed by a 15 min ramp to 100% MeCN). These data demonstrate in non-reducing conditions, **GP1** is largely stable over long periods of time, but can be quickly reduced under intracellularly relevant conditions.

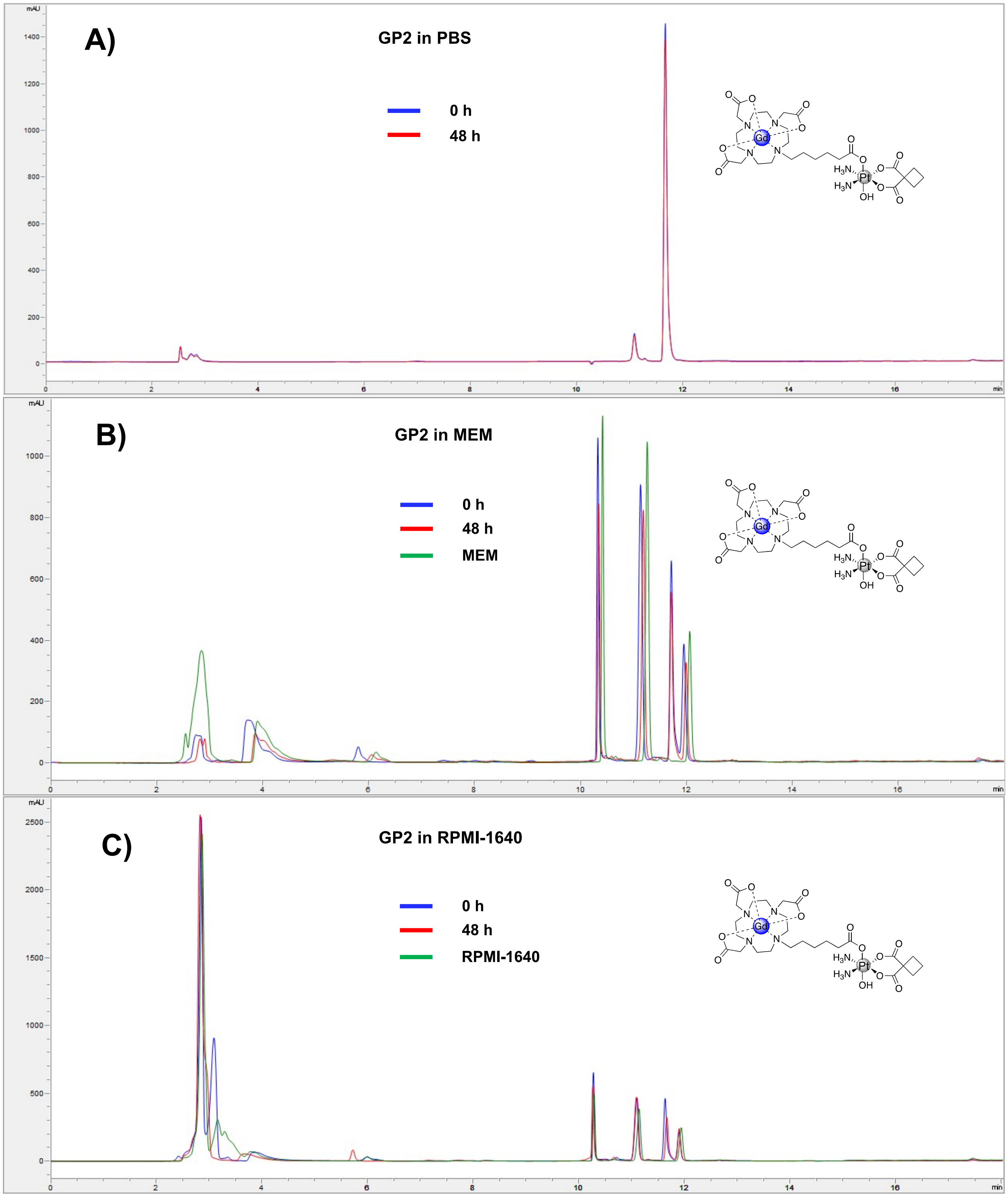
A)
GP2 in PBS
0 h
48 h
B)
GP2 in MEM
0 h
48 h
MEM
C)
GP2 in RPMI-1640
0 h
48 h
RPMI-1640
mAU
min

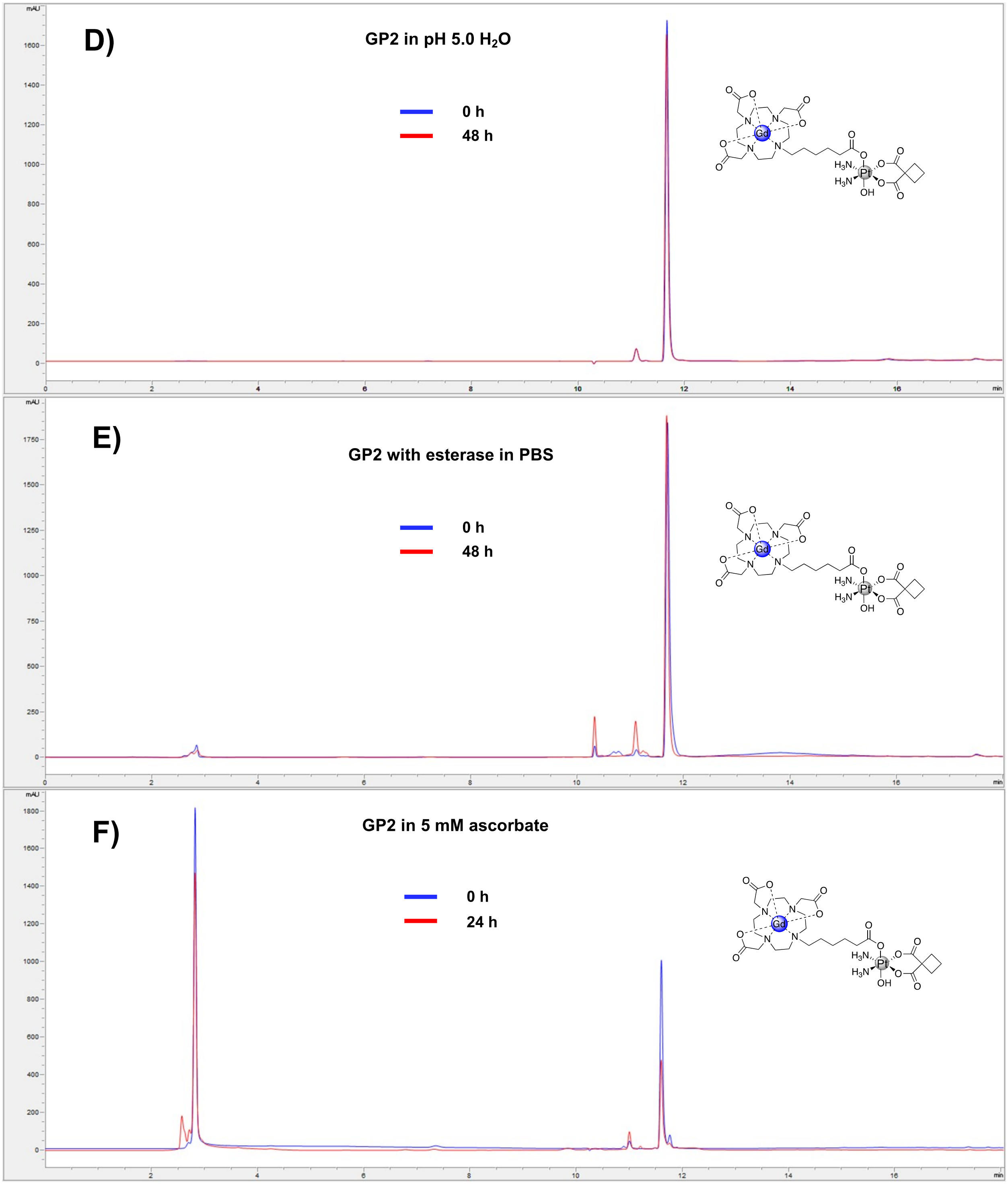
D)
GP2 in pH 5.0 H_2O
0 h
48 h
E)
GP2 with esterase in PBS
0 h
48 h
F)
GP2 in 5 mM ascorbate
0 h
24 h
mAU
min

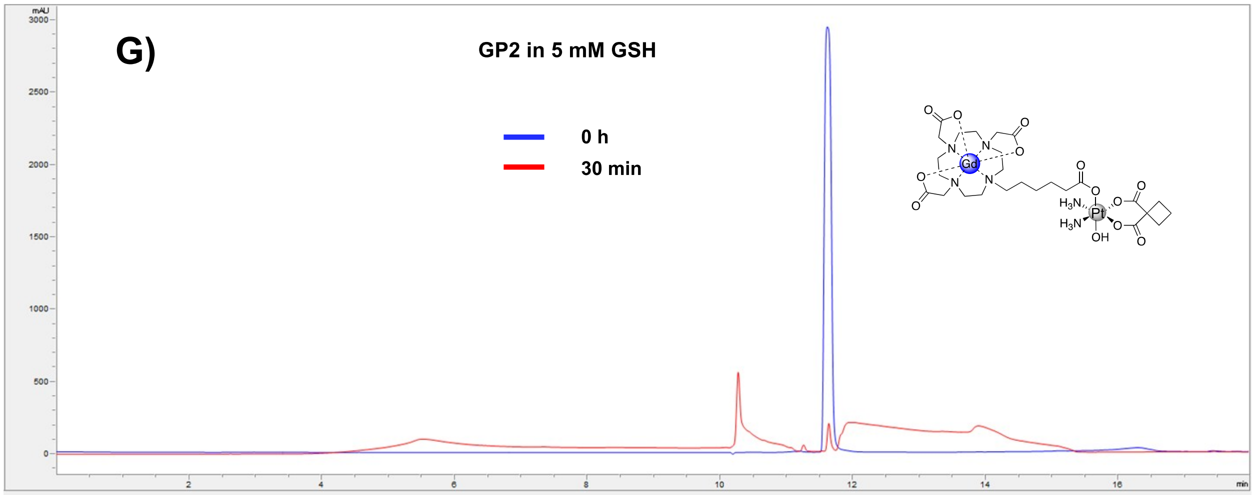

Fig. S4 Analytical HPLC chromatograms of **GP2** in (A) PBS over time (B) MEM over time (C) RPMI-1640 over time (D) pH 5 H_2O over time (E) PBS with porcine liver esterase (PLE) over time (F) 5 mM ascorbate over time and (G) 5 mM glutathione (GSH) over time. All HPLC runs used the same method (0-3 min 100% H_2O followed by a 15 min ramp to 100% MeCN). These data demonstrate that like **GP1**, **GP2** is largely stable over long periods of time in non-reducing conditions, but can be quickly reduced under intracellularly relevant conditions.

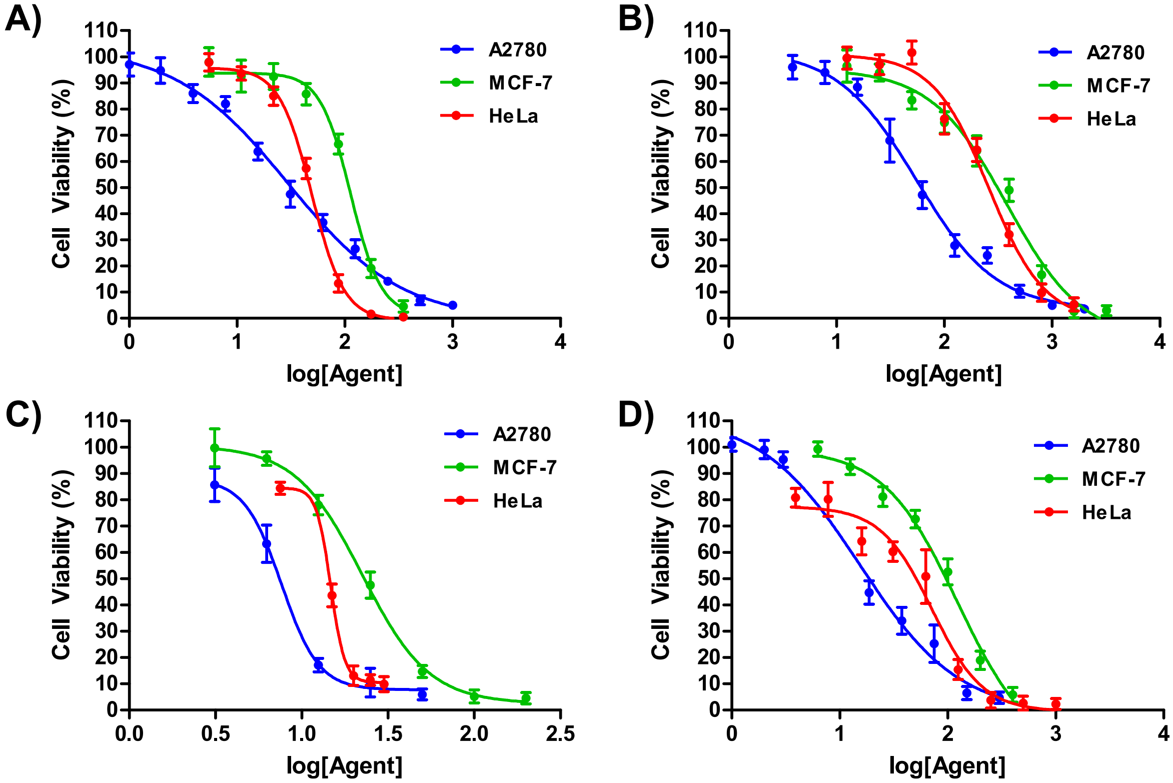

Fig. S5 (A) IC$_{50}$ curves of **GP1** in A2780, MCF-7, and HeLa cells. (B) IC$_{50}$ curves of **GP2** in A2780, MCF-7, and HeLa cells. (C) IC$_{50}$ curves of cisplatin in A2780, MCF-7, and HeLa cells. (D) IC$_{50}$ curves of carboplatin in A2780, MCF-7, and HeLa cells.

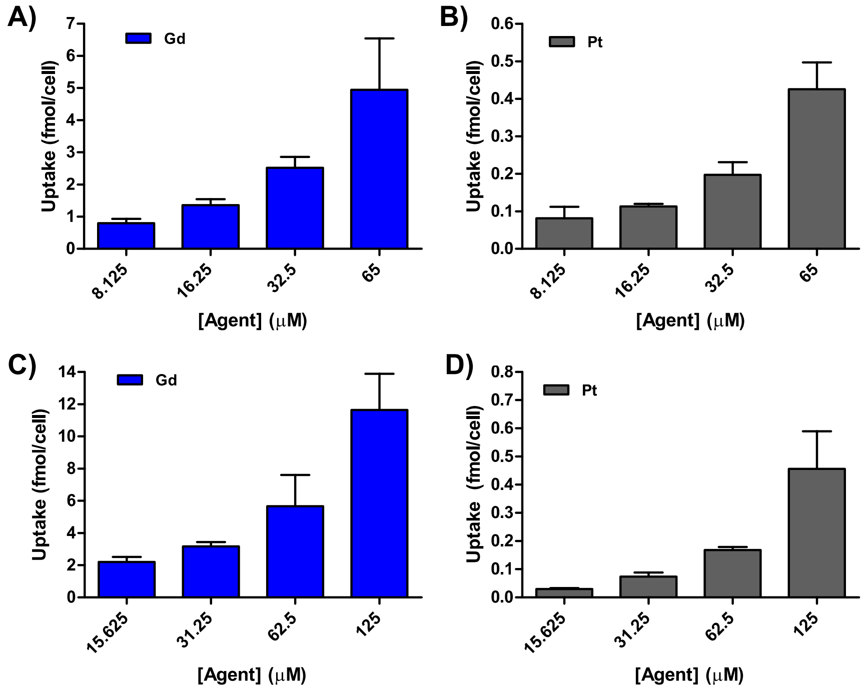

Fig. S6 (A) Accumulation of Gd in A2780 cells dosed with varying concentrations of **GP1** for 24 h. (B) Accumulation of Pt in A2780 cells dosed with varying concentrations of **GP1** for 24 h. (C) Accumulation of Gd in HeLa cells dosed with varying concentrations of **GP1** for 24 h. (D) Accumulation of Pt in HeLa cells dosed with varying concentrations of **GP1** for 24 h.

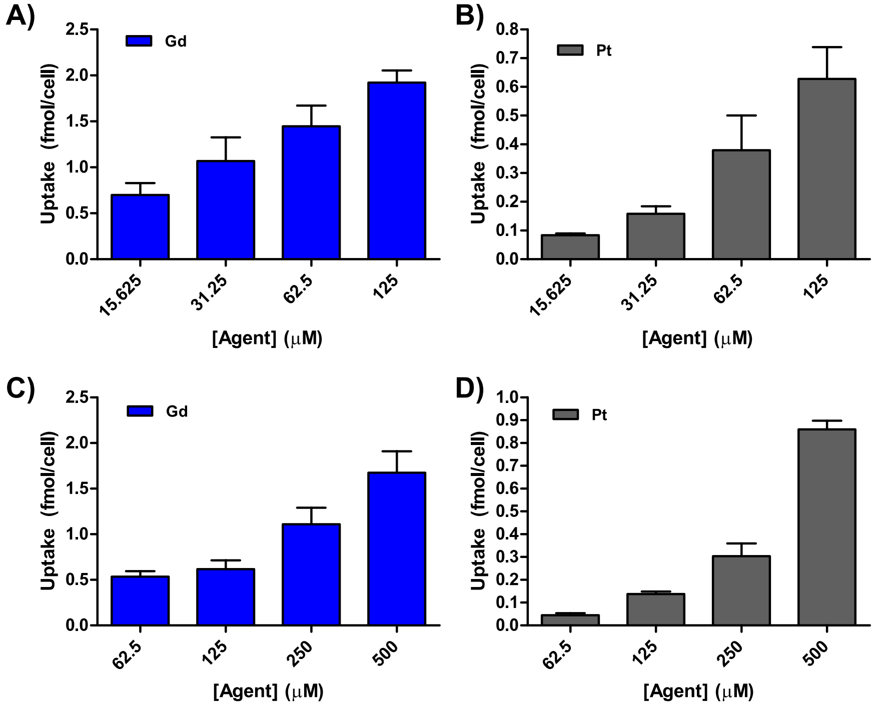

Fig. S7 (A) Accumulation of Gd in A2780 cells dosed with varying concentrations of **GP2** for 24 h. (B) Accumulation of Pt in A2780 cells dosed with varying concentrations of **GP2** for 24 h. (C) Accumulation of Gd in HeLa cells dosed with varying concentrations of **GP2** for 24 h. (D) Accumulation of Pt in HeLa cells dosed with varying concentrations of **GP2** for 24 h.

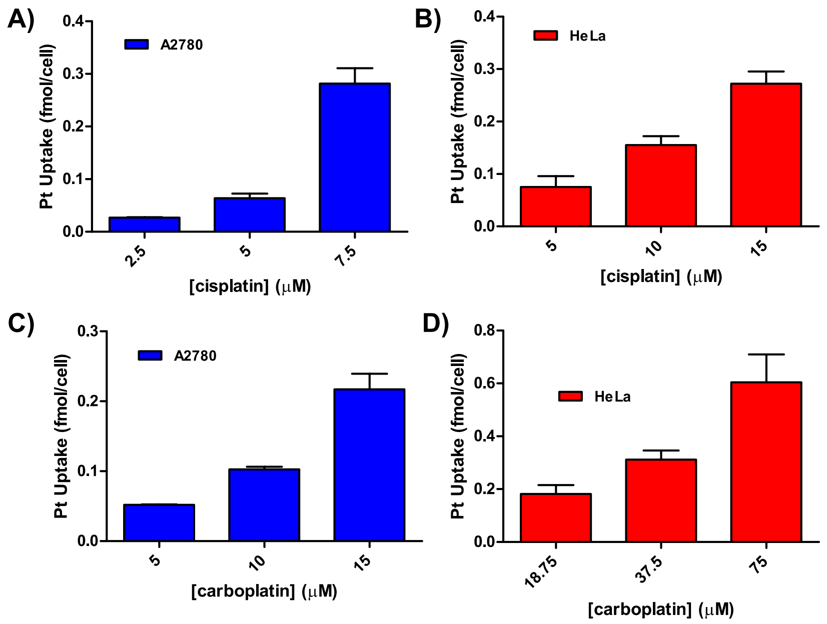

Fig. S8 (A) Accumulation of Pt in A2780 cells dosed with varying concentrations of cisplatin for 24 h. (B) Accumulation of Pt in HeLa cells dosed with varying concentrations of cisplatin for 24 h. (C) Accumulation of Pt in A2780 cells dosed with varying concentrations of carboplatin for 24 h. (D) Accumulation of Pt in HeLa cells dosed with varying concentrations of carboplatin for 24 h.

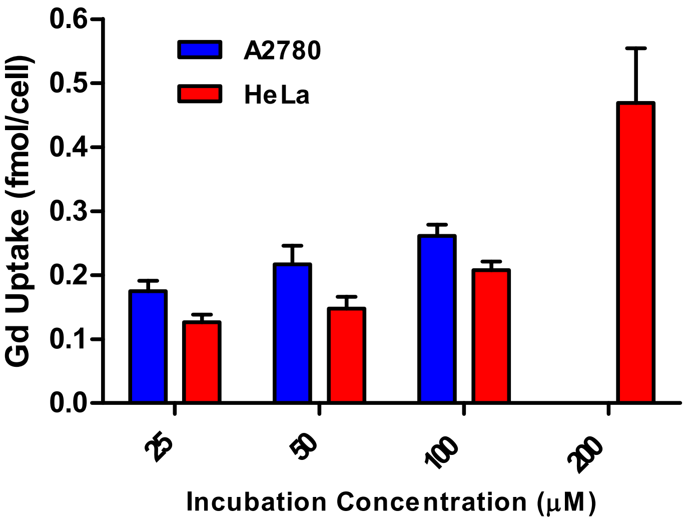

Fig. S9 Concentration dependent uptake of complex **1** in A2780 and HeLa cells after 24 hours. At incubation concentrations similar to **GP1** and **GP2**, **1** had significantly lower accumulation in both A2780 and HeLa cells. This supports that the Pt(IV) complexes are responsible for the higher uptake of **GP1** and **GP2** compared to complex **1**.

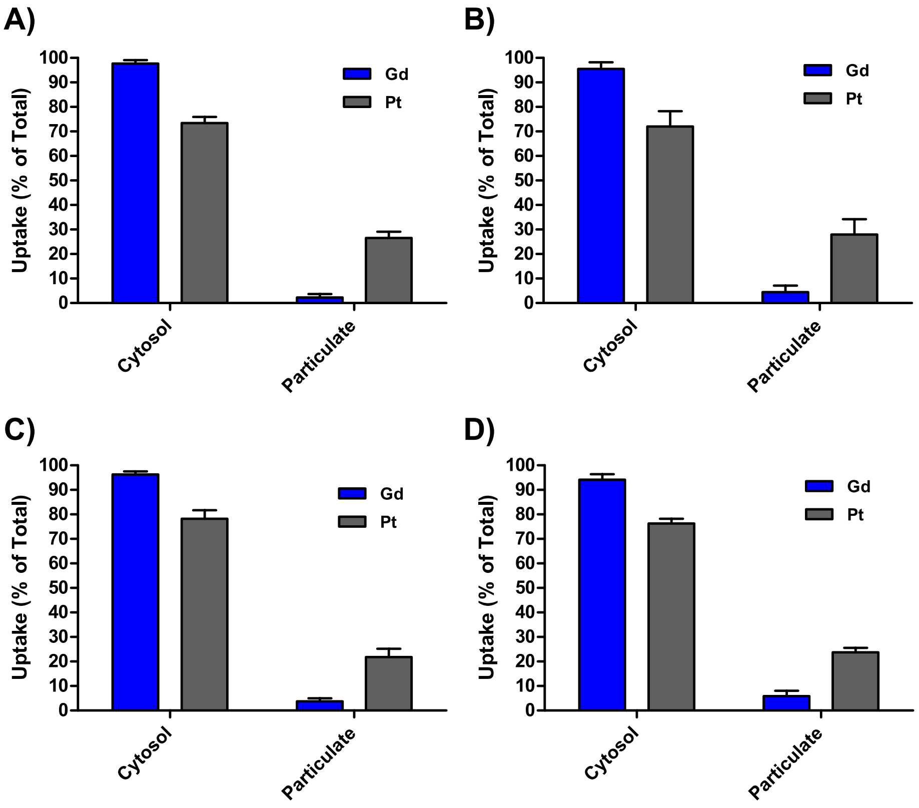

Fig. S10 Subcellular localization of Gd and Pt in cytosolic and particulate (membrane, organelles, cytoskeleton) cellular fractions. (A) A2780 cells incubated with 32.5 μM **GP1** for 24 h. (B) HeLa cells incubated with 50 μM **GP1** for 24 h. (C) A2780 cells incubated with 50 μM **GP2** for 24 h. (D) HeLa cells incubated with 250 μM **GP2** for 24 h. In all cases, ≥94% of Gd localized in the cytosol. The majority of Pt localized in the cytosol, but significant amounts were also found in particulate fractions. The differences in subcellular localization between Gd and Pt supports that **GP1** and **GP2** dissociate intracellularly.
